# Pharmacovigilance and bioethics: the importance of a neglected relationship

**DOI:** 10.3389/fdsfr.2025.1698515

**Published:** 2025-12-17

**Authors:** Giuseppe Alvaro, Michael Müller, Richa Chhibber, Robert Fuentes

**Affiliations:** 1 Institute of Pharmaceutical Sciences, Albert-Ludwigs-Universität, Freiburg, Germany; 2 Independent drug safety expert, London, United Kingdom; 3 Drug InfoNomics, LLC, Sarasota, FL, United States

**Keywords:** pharmacovigilance, bioethics, autonomy, beneficence, non-maleficence, justice, drug safety, public health

## Abstract

This study examines the critical, yet often neglected, link between bioethics and pharmacovigilance—disciplines that collectively aim to ground drug therapies in robust evidence and respect for patient rights. Despite their shared mission, scholarly dialogue between these fields remains sparse. This gap is bridged by tracing their historical foundations and addressing key ethical challenges in pharmacovigilance through the lens of four core bioethical principles: autonomy, beneficence, non-maleficence, and justice. For each principle, pivotal questions and illustrative examples are outlined, offering guidance on embedding ethical reasoning into pharmacovigilance practice. It is argued that while regulations, standard operating procedures, and digital tools are indispensable, they cannot alone resolve the ethical dilemmas posed by rapid scientific innovation, resource constraints, and global inequities, and they may reduce safety professionals to mere task executors. Consequently, it is imperative that alongside technical and regulatory progress, pharmacovigilance fosters the study and application of ethical principles in every aspect of its operation. This transformation will recast it from a purely technical function into an equitable guardian of public health, ensuring sound decision-making, promoting public trust, and delivering maximal benefit while upholding human dignity worldwide.

## 1 Introduction

Bioethics and pharmacovigilance are modern disciplines that have rapidly developed in the second half of the 20th century alongside the rapid expansion of scientific and technical knowledge applied to medicine. This is not accidental: while medical advances have indeed enabled the treatment of once incurable diseases, they have also raised ethical questions and introduced unprecedented risks to human health that must be rigorously evaluated and managed.

Both bioethics and pharmacovigilance aim to ensure that drug treatment and new drug development are based on the integrity of scientific evidence, proper evaluation of the benefit–risk ratio, and respect for patient rights. Based on these premises, a rich production of articles and books investigating the relations and potential synergies between these two disciplines might be expected, but this is not the case. One of the most important texts written on ethical issues raised by drug research, the *International Ethical Guidelines of the Council for International Organizations of Medical Sciences* (CIOMS, 2016), merely mentions the importance of safety (i.e., drug safety and pharmacovigilance) without further elaboration. Similarly, the term “drug safety” is mentioned only once and in passing by the monumental *Encyclopedia of Bioethics* (Post, 2004). Lastly, a literature search conducted in PubMed on 17 October 2025 using the terms “bioethics” and “pharmacovigilance,” without applying temporal, geographical, or other restrictive filters, identified 28 articles. However, none of the retrieved publications provided an overarching analysis of the conceptual intersections and practical implications linking bioethics and pharmacovigilance. The aim of the present study is to help address this gap by, first, elucidating the ethical foundations that underpin the mission of pharmacovigilance—foundations often eclipsed by regulatory and operational priorities—and second, by an examination of how core bioethical principles can inform and guide the design, implementation, and interpretation of pharmacovigilance activities.

### 1.1 History and role of pharmacovigilance

The recognition that therapeutic agents may pose risks alongside benefits has deep historical roots. As early as the ninth century CE, Arab authorities implemented drug quality controls and penalized counterfeiters. By the tenth century, the Salerno Medical School in Italy identified harmful substances, and in 1224, Holy Roman Emperor Frederick II mandated routine drug inspections, with capital punishment for pharmacists implicated in fatal outcomes (Davies, 1999).

The modern era of pharmacovigilance was catalyzed by the thalidomide tragedy in the early 1960s. Marketed in Germany in 1956 as a sedative and treatment for morning sickness, thalidomide was considered safer than barbiturates and was rapidly adopted in over 50 countries. However, by 1959, cases of phocomelia—a rare congenital malformation—emerged in Germany, with global incidence rising sharply. The United States was spared due to FDA reviewer Frances Kelsey, who withheld approval, citing insufficient safety data (Bren, 2001). Her concerns were validated in 1961 when Australian obstetrician William McBride linked thalidomide to birth defects in a letter to *The Lancet* (McBride, 1961). Subsequent clinical and epidemiological data confirmed the association, leading to the drug’s withdrawal. An estimated 12,000 infants were affected, with a 40% mortality rate (Lenz, 1988; Matthews and McCoy, 2003). This event prompted the first comprehensive drug safety regulations worldwide.

The term *pharmacovigilance*, derived from Greek *ϕάρμακον* (drug or poison) and Latin *vigilare* (to be vigilant), was introduced in the 1970s by French pharmacologists to describe the systematic assessment of adverse drug effects (Bégaud et al., 1994). It has since gained widespread acceptance in scientific and regulatory contexts, although the term “drug safety” remains common in English-speaking regions. The World Health Organization defines pharmacovigilance as “…the science and activities relating to the discovery, evaluation, understanding and prevention of adverse effects or any other possible drug-related problems” (WHO, 2006). The European Commission further emphasizes its public health role: “Pharmacovigilance is the process and science of monitoring the safety of medicines and taking measures to reduce their risks and increase their benefits. It is a key public health function” (European Commission, 2025). This definition underscores the discipline’s societal relevance, encompassing risk identification, communication, and management throughout a drug’s lifecycle. Effective pharmacovigilance requires coordinated efforts among patients, clinicians, researchers, industry stakeholders, and regulatory authorities.

### 1.2 History and role of bioethics

In the *Encyclopedia of Bioethics*, Reich defines bioethics as “…the systematic study of the moral dimensions—including moral vision, conduct, and policy—of the life and health sciences, using various ethical methodologies and with an interdisciplinary focus” (Reich, 1995; Post, 2004). The term originates from the Greek *ήθος* meaning the moral habits of a person or community, and *βíος*, meaning life. It was first introduced by German theologian Fritz Jahr in 1927, who, inspired by Kant’s categorical imperative, proposed a “bioethical imperative” whereby all living beings deserve respect and should be treated as ends rather than means (Lolas, 2008). The term gained prominence in the 1970s through the work of US oncologist Van Rensselaer Potter (1971), who advocated for a discipline that integrated scientific and humanistic knowledge to safeguard humanity amid rapid biomedical advances. Around the same time, André Hellegers established the Kennedy Institute for the Study of Human Reproduction and Bioethics in Washington, D.C.— the first dedicated bioethics institute. Foundational texts emerged during this period, including the *Encyclopedia of Bioethics* (Post, 2004), the Declaration of Helsinki (World Medical Association, 2024), and the Belmont Report (National Commission, 1979).

As bioethics evolved globally, two primary research strands emerged: one focused on medicine and biomedical sciences, and another encompassing broader scientific and technological domains. Each is shaped by distinct theoretical frameworks and underlying worldviews (*Weltanschauungen*). The principal models are presented in Table 1, drawn from the conceptual frameworks and classifications proposed by Sgreccia (1999) and Zeppegno (2020) in their respective contributions to bioethics.

**TABLE 1:** Comparative overview of the main bioethical models.

Model	Core principle	View of morality	View of personhood	Key exponents
Socio-biological	Morality evolves like biological traits, shaped by societal development	Functional and adaptive; values change with societal evolution	A product of evolutionary forces	Edward Osborne Wilson
Liberal–radical subjectivist	Individual autonomy and freedom are the sole moral references	Morality is subjective; no objective or transcendent values	Defined by autonomous choice	Karl Popper, Herbert Marcuse, Uberto Scarpelli
Utilitarian contractualist	Maximize pleasure, minimize pain for the majority	Public ethics based on cost–benefit and subjectivism of the majority	Defined by functional qualities such as autonomy and relational capacity, grounded in intersubjective agreement	Peter Singer, Hugo Tristram Engelhardt
Ontologically grounded personalism	Every human is a person with inalienable dignity from conception to natural death	Morality rooted in intrinsic human dignity and solidarity	Defined by substantive personhood, not functional capacity	Elio Sgreccia

**FIGURE 1:**
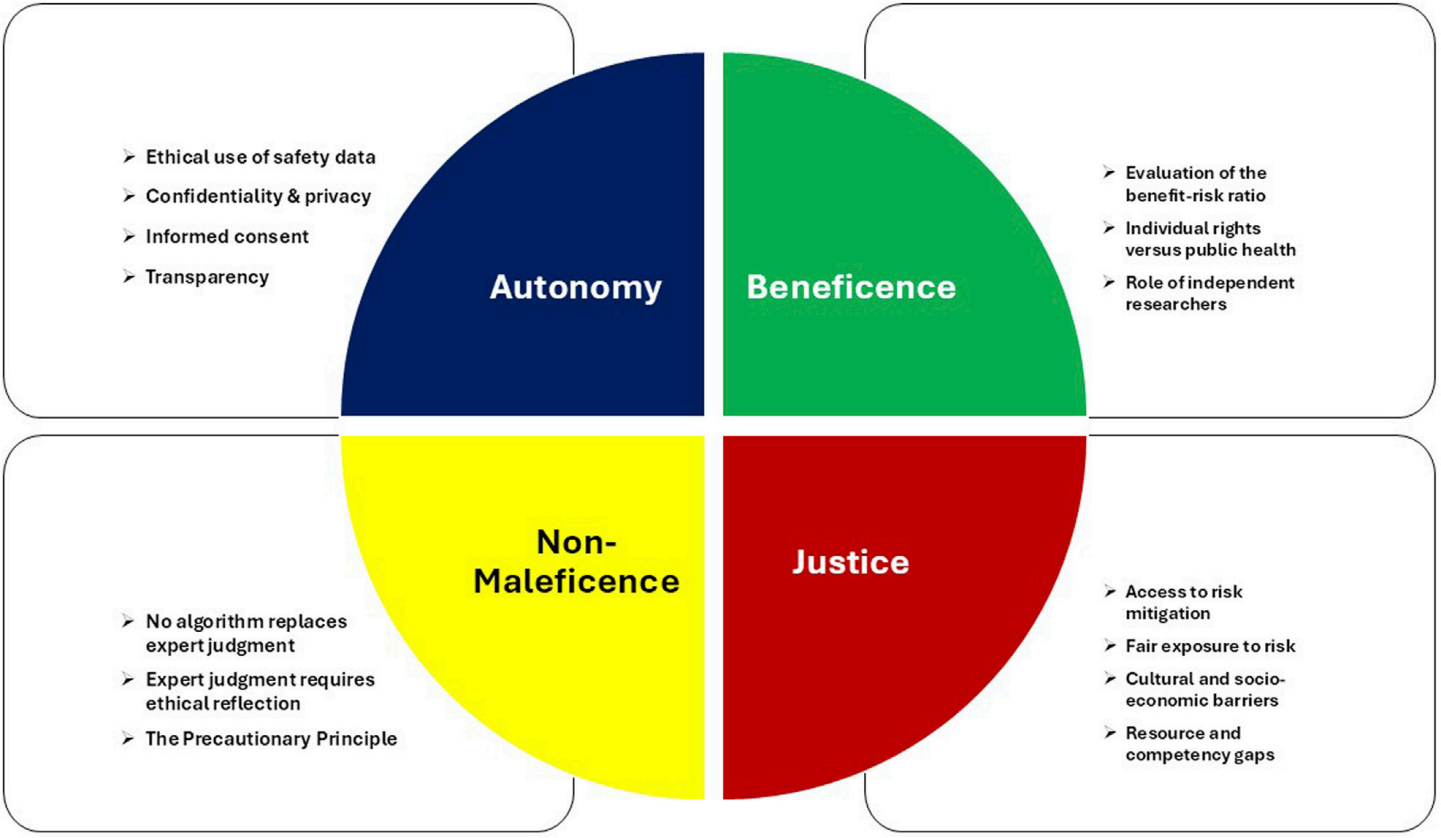
The four bioethical principles and their implications for pharmacovigilance.

An additional model prevalently used in medicine is principlism, which stems from the work of Tom Lamar Beauchamp and James Franklin Childress. In their book *Principles of Biomedical Ethics*, they propose a set of four ethical principles to be considered in medical research and healthcare practice (Beauchamp and Childress, 2019). Although it is not without its weaknesses—chiefly the absence of a theoretical framework and a hierarchy among the proposed principles—the model’s relative simplicity and pragmatic orientation have contributed to its widespread adoption in healthcare, particularly in Anglo-Saxon contexts, and make it a suitable starting point for exploring shared concerns in bioethics and pharmacovigilance. The four principles are those of autonomy, beneficence, non-maleficence, and justice (Figure 1). The following sections individually examine these principles and their relevance for pharmacovigilance.

## 2 Autonomy and its ethical relevance in pharmacovigilance

The concept of autonomy, from the Greek *αùτóς* (self) and *vòmos* (law), originally referred to the self-governance of city-states. In bioethics, it denotes an individual’s capacity for self-determination—the ability to make informed decisions and act freely according to personal values. This capacity develops over time and may be constrained by illness, socioeconomic conditions, or institutional restrictions. Since the Nuremberg Code, respect for autonomy has been a cornerstone of biomedical ethics (British Medical Journal, 1996). Individuals with diminished autonomy such as children, incarcerated persons, or those with cognitive impairments require special protections. Historically, medical practice often prioritized beneficence over autonomy, fostering a paternalistic model that is now widely challenged. However, Beauchamp and Childress (2019) emphasize that ethical principles, including autonomy, are not absolute and must be balanced against other ethical standards when moral conflicts arise. This tension was evident during the COVID-19 pandemic, where individual autonomy (e.g., vaccine refusal) conflicted with public health imperatives. Ethical discourse increasingly highlights the need to pair freedom with responsibility and solidarity, recognizing the interdependence of individuals within society (Dawson and Jennings, 2012). Nonetheless, the protection of life remains a paramount ethical obligation, justifying temporary restrictions on personal liberty during public health emergencies—provided such measures are proportionate and time-limited (European Group on Ethics in Science and New Technologies, 2020). Respect for patient autonomy is a foundational principle in pharmacovigilance, a discipline that entails the collection and analysis of extensive personal data, transparent communication of risk, and the active, informed engagement of patients in decisions concerning their health.

### 2.1 Ethical use of pharmacovigilance data

Historically, there were essentially two sources of information regarding the undesirable effects of drugs in humans: clinical trials conducted during the development of new drugs, and spontaneous post-marketing reports made by patients and healthcare professionals during clinical practice. More recently, longitudinal healthcare databases, such as electronic health records (EHR), are also increasingly used to provide insights into the safety of drugs (Star et al., 2015; Davis et al., 2023; Kim et al., 2024). In all these cases, the information provided (including personal data such as initials, age, weight, and medical history) is collected in huge, computerized databases and is processed mainly by pharmaceutical companies, healthcare organizations and health authorities, according to the processes and timing imposed by regulations and the degree of development of each national healthcare system. In the member states of the European Union and other highly developed countries, some of the personal information collected is considered particularly sensitive and is protected by law because it concerns the patient’s race, ethnicity, biometric and genetic data, or gender and sexual orientation (ENCePP, 2025). What about elsewhere? It quickly becomes apparent that where there are no applicable laws and standards of reference, there is a need for a code of pharmacovigilance ethics that defines the obligations ensuring the ethical use of data collected during clinical or epidemiological studies, and in clinical practice. In observing the principle of autonomy and, more generally, of respect for the patient, these obligations should include the following as a minimum:

- An obligation to tell those who report or provide pharmacovigilance information (e.g., regarding undesirable effects) who, how, for what purpose, where, and how long it will be stored.
- The obligation to use the information received consistently with the stated purpose and not to share it with third parties except for that purpose (e.g., with health authorities).
- The obligation to limit the personal information collected, especially if it is sensitive, to the minimum necessary, and to take all necessary technical and operational measures to protect it from accidental or criminal breach.
- The obligation to provide the name and contact information of the office in charge of controlling the stored data, along with information regarding the patient’s rights to know, to rectify, and, where applicable, to delete their data.

### 2.2 Confidentiality and privacy

If we describe “patient autonomy” as the space over which patients can exercise their right to self-determination, then confidentiality and privacy can be considered the necessary safeguards of this space by limiting access to it. Sometimes used as synonyms, “confidentiality” and “privacy” represent different concepts, depending on the nature of the relationship between the parties sharing the information—whether such sharing took place “in confidence” or not. For example, a cyber-attack accessing a hospital’s medical records violates patients’ privacy but not confidentiality, since no covenant or agreement exists between the hacker and the patients. Conversely, a pharmaceutical company or health authority that discloses personal, clinical, or safety information provided by a patient also violates the confidentiality of the relationship, just like a doctor who carelessly discloses data protected by patient confidentiality. This has profound ethical and practical implications, as concerns about the security of healthcare data may undermine public trust, prompt patients to withhold sensitive information, and pose governance challenges—particularly in developing countries (Bani Issa et al., 2020; Layman, 2020; Gesicho et al., 2017). Some basic precautions taken in pharmacovigilance to ensure data confidentiality and patient privacy include anonymizing collected data, controlled sharing limited only to insiders (e.g., health authorities), and adopting robust IT systems that are resistant to breach attempts. However, ill-defined gray areas remain. One such area concerns the use of data regarding adverse drug effects posted by the billions of users of social platforms such as Facebook, X, and the like. Despite the many limitations related to the quality and completeness of these data, there is no doubt that they can provide useful information for the early identification of possible undesirable effects, and therefore it is ethically justified to analyze and evaluate them—think, for example, of possible reports of suicidal ideation following the use of a drug. The fact that this information is voluntarily shared suggests tacit consent to read it but not to use it—whether for pharmacovigilance, insurance, or other purposes (Azam, 2018). This interpretation is confirmed by the results of a study that found that only 34% of those surveyed agreed with the use of their published data for pharmacovigilance purposes, while 90% believed that this was permissible only with explicit consent (Omar and Harris, 2016).

### 2.3 Informed consent

The earliest formal articulation of consent in clinical research was established in 1947 by the Nuremberg Code, following the trial of Nazi physicians convicted of conducting inhumane experiments on human subjects. In its first article, the code introduced the principle of mandatory “voluntary consent,” stipulating that individuals enrolled in clinical trials must provide consent freely—without coercion, fraud, or deception—and with sufficient understanding of the study’s nature, duration, purpose, risks, and potential inconveniences (British Medical Journal, 1996). This foundational ethical construct, now universally referred to as “informed consent,” has become central to clinical research and is consistently emphasized across major international guidelines, including the Declaration of Helsinki, Good Clinical Practice (GCP), and the International Ethical Guidelines, which are reflected in national regulations. Waivers to the requirement for informed consent are permitted only under specific circumstances, such as emergency settings or trials involving investigational products expected to pose only minimal risk (WMA, 2024; ICH E6, 2025; CIOMS, 2016). A core obligation of informed consent is the clear communication of risks and uncertainties inherent in the study protocol, along with the measures in place to prevent and mitigate them—an essential function of pharmacovigilance that remains challenging to implement in practice. A first order of difficulty involves patients who are not or only partially autonomous (e.g., children and patients with cognitive deficits or those in immediate danger of death). In these cases, reference is usually made to the role of legal guardians and ethic committees to make decisions on behalf of the patient based on criteria that Beauchamp and Childress (2019) classify as the “substituted judgment standard,” the “pure autonomy standard,” and the “best interest standard.” A second, more insidious and frequent level of difficulty is related to the objective difficulty of comprehensibly communicating the risks associated with an experimental drug or study protocol to patients who, due to cultural, intellectual, or psychological limitations (e.g., emotional stresses related to the underlying disease), are unable to understand them (Bester et al., 2016). For example, in one oncology study, 90% of participants stated that they were satisfied with the information provided with the informed consent form. However, upon a check of their level of understanding, approximately 75% of them showed that they did not understand that the study involved experimental therapies of unproven efficacy and safety. An additional 25% did not realize that the primary goal of the study was to develop therapies that would be useful to future patients more than to themselves (Joffe and Cook, 2001). Similar findings and concerns have been echoed in more recent studies (Bergenmar et al., 2008; Falagas et al., 2009). In the face of these difficulties, the most recent guidelines reiterate that “The person obtaining consent must ensure that the potential participant has adequately understood the information provided” (CIOMS, 2016), yet no method of verification (e.g., use of questionnaires) is suggested, much less recommended. Finally, an additional difficulty concerns the need to avoid generic and standard summaries in the information provided to the patient but instead trying to personalize the message as much as possible, taking into account the patient’s personal, cultural, and religious beliefs. This may mean providing information important to the individual patient even when it is not of general interest, such as details concerning the mechanism of action of contraceptive methods, the intended use of genetic or biological material, or the origin (e.g., embryonic) of an experimental drug. Similar considerations to those raised in the context of clinical research remain valid when informed consent is sought in clinical practice—for example, before performing surgical, endoscopic, or radiological procedures, or treating patients with drugs that pose high risks to their health or that of their offspring. Crucially, both during clinical trials and in clinical practice, it should be ensured that obtaining informed consent is not reduced to a hastily extorted signature or a disclaimer limiting the physician’s responsibilities but represents a true moment of encounter and dialogue between physician and patient. In addition to ensuring respect for the patient, properly obtained informed consent is likely to foster therapeutic communication and alliance between physician and patient, adherence to the treatment regimen, and even healing (Dowd, 1996).

### 2.4 Transparency of information

The benefit–risk ratio of a drug is never fully established during clinical trials due to the inherent limitations of research—for example, limited number of patients studied and short duration of studies. In addition, it is not uncommon for additional benefits or risks to be discovered years or decades after the drug is launched on the market. An example is aspirin, which—synthesized in 1897—was recognized as useful in preventing coronary thrombosis 53 years later (Miner and Hoffhines, 2007), while it took 39 years to realize that it could cause gastric bleeding and another 20 years for this information to become public knowledge (Davies, 1999).

Thus, to ensure patient autonomy, the one-time signing of informed consent is not enough; it is necessary that ongoing knowledge updates regarding drug benefits and risks be communicated in a transparent and timely manner throughout the time the drug is used, from phase I clinical trials to withdrawal from the market. For this reason, the European Medicines Agency (EMA) publishes online the detailed European Public Assessment Reports (EPARs) that lead to the authorization or non-authorization of drugs for marketing in EU member states, as well as any updates to them (EMA, 2023). Transparency of action is also strongly promoted by the US FDA and health authorities in other countries. There is no doubt that this approach promotes informed use and trust by physicians and patients (Waller, 2011). However, unrestricted transparency is neither possible nor desirable, not only because of the limits posed by privacy and intellectual property protection but also because it is counterproductive. For example, the premature communication of drug risks suggested by inconclusive evidence may generate unnecessary anxiety in patients or even cause them to unnecessarily discontinue life-saving therapies (Callréus, 2013). Furthermore, it has been shown that the communication of openly divergent and conflicting views among experts, while legitimate and functional for scientific progress, undermines public confidence in the rationality of decisions made (Bouder, 2011), so the presentation of different viewpoints must be appropriately managed to avoid responses dangerous for public health.

## 3 Beneficence and its ethical relevance in pharmacovigilance

The second principle proposed by Beauchamp and Childress (2019) is that of beneficence, from the Latin *beneficentia*, meaning “to do good.” It is a principle underlying the practice of medicine, which finds in it its purpose and *raison d'être*, as well as all those disciplines and activities—including pharmacovigilance—that aim to promote and safeguard public health. They use this term in the broadest sense “…to include all rules, regulations, and actions intended to benefit or promote the welfare of other people,” rather than in the sense of the utility dear to utilitarians, which assumes balancing benefits, risks, and costs to produce the best overall outcomes (Beauchamp and Childress, 2019). However, in the case of pharmacovigilance and other public health disciplines, it is this narrower meaning—the assessment of benefits and risks—that matters.

### 3.1 Evaluation of the benefit–risk ratio

As stated in the Declaration of Helsinki, “Medical research involving human subjects may be conducted only if the importance of the objective outweighs the risks and burdens to the research subjects. All medical research involving human subjects must be preceded by a careful assessment of the foreseeable risks and burdens to the people involved in the research, relative to the foreseeable benefits to them and to other people affected by the condition being researched” (World Medical Association, 2024). The goal of new drugs development is thus to demonstrate that their use has more benefits than risks—that is, that their benefit–risk ratio is favorable. Pharmacovigilance plays a critical role in this process by identifying, assessing and managing as soon as possible all drug risks—especially the serious ones—taking into consideration the expected benefits of their use. Only once this is done is it possible to draw conclusions about the benefit–risk profile. However, this process is far from simple and raises important ethical questions: who and how decides whether the benefit–risk ratio of a drug is favorable? In other words, when is it permissible to conclude that a certain drug does more good than harm? Moreover, how much and how do we weigh the benefits and risks to the individual patient and those to the community? Who decides is clear: the health authorities of the various countries who are responsible for promoting and protecting public health. However, although this decision-making role of health authorities is generally respected, there is no shortage of examples of criticism and protests by those who have not felt adequately represented and protected by them. Among these was the AIDS patients’ protest on 11 October 1988, when activists from the ACT UP organization occupied the FDA to protest the slowness and caution that had hitherto characterized clinical research for new drugs against the human immunodeficiency virus. As a result of the protests, the FDA changed the criteria and procedures for approving clinical trials, accelerating the development of new and effective therapies (CATO Institute, 2020). Additional examples include the controversy in the 1990s regarding FDA approval of breast implants (Angell, 1992) and the more recent demonstrations against mandatory vaccination and other public health measures enacted during the COVID-19 pandemic (BBC News, 2021; Erlanger, 2020). The possibility of disagreement over decisions made by health authorities is at least in part due to the objective difficulties in assessing the benefit–risk ratio, which in addition to scientific aspects must consider cultural, economic, and social considerations and values. In addition, there is no universally accepted method or criterion for determining when the benefit–risk ratio of a drug is favorable or unfavorable, and no one can decide incontrovertibly how many and what risks (e.g., number of deaths caused by a drug) are acceptable in relation to the expected benefits (e.g., number of lives saved by the same drug) —all the more so considering that the perspective of cured and harmed patients is understandably quite different. Current FDA and EMA guidelines regarding the evaluation of the benefit–risk ratio of drugs are predominantly based on the use of qualitative methods and expert opinion (EMA, 2011; FDA, 2021), while quantitative methods (often themselves based on arbitrary assumptions and thresholds) at best are used to provide additional insights but certainly not to make decisions (Curtin and Schulz, 2011). This absence of shared methodologies and standards is at the root of many discordant opinions and decisions among different health authorities, the medical community, and patients. This was evident, for example, in the case of contraception, which was imposed and later withdrawn some years ago by the EMA to prevent paternity in patients treated with mycophenolate (Damkier et al., 2016). Further differences of opinion may arise due to extra-scientific elements of evaluation, which need to be considered by health policymakers. These include the need to consider the relevant therapeutic context, the existence of alternative treatments, the magnitude of unknowns related to the limitations of clinical research, the perception of benefits and risks by patients and healthcare providers, and the availability of resources to devote to a particular therapy relative to the general needs of the community. Lastly, specific ethical challenges concern the assessment of the benefit–risk ratio in special situations or patient populations.

### 3.2 Rights of individuals and protection of the community

The earliest ethical precepts in medicine can be traced back to the Hippocratic Oath, which binds the physician to do good to the sick person as far as his ability and judgment allow (Edelstein, 1943). This basic approach to protecting the good of the individual reasserted itself as a central ethical theme after the infamous experiments conducted by Nazi doctors, in which thousands of human beings lost their lives in atrocious ways (Lifton, 1986). Much of that aberrant research aimed to elucidate the body’s response to several types of trauma, such as forced decompression, hypothermia, burns, amputations, and poisoning, as well as experimental therapies designed to mitigate them. This involved research aimed at developing potentially life-saving therapies and techniques for “Aryans” at the cost of sacrificing Jews, the disabled, and other human beings considered inferior. Unfortunately, despite the promulgation of the Nuremberg Code in 1947, grossly unethical experimentation continued in various nations, including the United States, until the 1970s. Among the best-known examples are:

- The study conducted in Guatemala by the US Public Health Service between 1946 and 1948, in which 1,300 people, including soldiers, orphans, prostitutes, and mentally ill subjects, were infected with syphilis, gonorrhea, and chancroid bacteria without their knowledge (Presidential Commission for the Study of Bioethical Issues, 2011).
- Tuskegee’s syphilis study, which in gathering information on the natural course of the disease, left hundreds of Black Americans without treatment, even after the discovery of penicillin (Brandt, 1978). The US Public Health Service also conducted this study.
- The experiments conducted by various US government agencies between 1945 and the 1970s on hundreds of patients deliberately exposed to radiation during atomic weapons testing or through the administration of radioactive isotopes—among them soldiers, pregnant women, and children with disabilities (Welsome, 1999).
- The deliberate administration of hepatitis virus to intellectually disabled children admitted to Willowbrook State Hospital in New York from 1965 to 1971, with the aim of understanding the modalities of transmission of the virus. All the children became ill with hepatitis, some in a severe form, in an experiment conducted without their knowledge and from which they could derive no benefit (Offit, 2007).

The examples given explain and justify the attention given to the rights of individuals by all the major bioethics texts of recent decades, from the Declaration of Helsinki to the International Ethical Guidelines. However, the recent COVID-19 pandemic and the imposition of public health measures restricting personal freedoms have led to the realization that alongside the rights of individuals, bioethics must also consider the impact of medical research and practice on the community. Among the characteristics of this “public health ethics” is that it aims to prevent and thus defend the state of health rather than treat the diseases, is concerned more with collective health than with the health of the individual, and influences activities and disciplines other than health, such as those that determine the regulatory and socioeconomic conditions that promote or hinder health (Dawson and Verweij, 2007). These same characteristics apply to pharmacovigilance, which while based on scientific data, must still confront ethical issues and inform balanced decisions that respect the rights of the individual and society. During clinical research, this balance must be sought, as in the case of children and women of childbearing potential who are not using highly effective contraceptive methods. These particularly vulnerable patient populations have historically been excluded from clinical trials for the ethical reasons of protecting them from the risks and unknowns of research. However, this approach has led to the use in clinical practice of drugs that have never been studied or approved for use in pregnancy and pediatrics. The off-label use of a medicinal product (i.e., not in accordance with the approved prescribing information) is far from a marginal phenomenon, since it affects at least one-third of all hospitalized children, up to 90% of infants in intensive care (Guidi et al., 2022), and 80–90% of pregnant women (Wesley et al., 2021; Laroche et al., 2020). This has led to a reconsideration of the ethical rationale behind the exclusion of these patients from clinical trials (Buchanan and Miller, 2006), balancing the need to protect the health of participants (protection of the individual) with the need to ascertain in a controlled setting the benefit–risk ratio of the drug in these patient populations (protection of the community). Furthermore, the *a priori* exclusion of pregnant women from clinical trials is now considered ethically unjustifiable because it violates the principles of autonomy, justice, and non-maleficence to their detriment (Zur, 2023).

Things get even more complicated in the case of drugs already on the market which are sometimes used by millions of people in dire need. A recent example is offered again by the COVID pandemic, which led to the rapid testing and authorization for emergency use of mRNA (messenger RNA) vaccines, based on a technology never authorized for use in clinical practice. In this case, pharmacovigilance played a critical role by not only generating data essential for public health protection while protecting clinical trial participants but also by addressing the uncertainties typical of clinical trials data—like the lack of long-term efficacy and safety data—while expediting the development of vaccines urgently needed by millions of patients worldwide. All of this was in the context of a public opinion more ready to fear the rare but sometimes fatal undesirable effects reported (sometimes tendentiously) in the press than the enormous benefits offered to the population as a whole. The “perfect storm” brought about by the COVID-19 pandemic is exemplary of the situation described in 2013 by Callréus: “Pharmacovigilance of marketed drugs is often conducted in circumstances characterized by unknowns and urgencies, the former due to the very nature of the available data and the latter by media attention and the fact that large segments of the population are exposed to the suspect drug. Therefore, ethical principles applied during clinical research may not be readily applicable after marketing authorization” (Callréus, 2013). Despite this caveat, ethical studies on the problems associated with the regulation of pharmaceuticals and vaccines continue to be particularly scarce in the very case of drugs authorized for use in clinical practice (Thompson et al., 2014).

## 4 Non-maleficence and its ethical relevance in pharmacovigilance

The principle of non-maleficence, an ethical imperative to avoid causing harm, is foundational to both medical practice and pharmacovigilance. Often encapsulated in the Latin maxim *primum non nocere* (“First, do no harm”), it remains a cornerstone of medical ethics and a guiding principle in drug safety, where the primary objective is to minimize preventable harm associated with pharmacological interventions.

This imperative is particularly salient today, as iatrogenic harm continues to rank among the leading global causes of morbidity and mortality (Jha et al., 2013). Adverse drug reactions (ADRs) account for a substantial portion of this burden, contributing to approximately 15% of hospital expenditure (Slawomirski et al., 2017) and affecting an estimated 17% of hospitalized patients (Miguel et al., 2012). A meta-analysis of 70 clinical trials further highlights the severity of the issue, identifying drug-related harm as the predominant source of preventable patient injury, with 12% of cases classified as serious or fatal (Panagioti et al., 2019).

In this context, risk assessment must be carefully balanced against anticipated therapeutic benefits. While the use of potentially toxic drugs may be justified in treating life-threatening conditions—for example, chemotherapeutics in oncology—minor adverse effects may be deemed unacceptable when managing mild diseases, particularly when safer alternatives are available.

### 4.1 Identification and management of drug risk

As mentioned earlier, pharmacovigilance aims to minimize drug risks by identifying and managing them as early and effectively as possible, considering the expected benefits of treatment. This occurs from the first dose used in phase I clinical trials, which are conducted on a very limited number of healthy volunteers monitored around the clock using very low doses of the drug and taking all precautions suggested by the knowledge accumulated during preclinical studies and experience with drugs of the same class (if any). Later clinical trials identify the optimal dose and define the benefit–risk profile of the drug in increasingly larger patient populations. Despite the rigor and integrity of this process, clinical trials do not allow for the comprehensive evaluation of the safety profile (and thus the benefit–risk ratio) of new medicines because of their well-known inherent constraints. These include the limited number of subjects enrolled (usually less than 3000, and sometimes a few hundred or even dozens in the case of rare diseases), their short duration (usually not more than 1 year), as well as the narrow selection criteria that exclude from the studies many situations present in clinical practice, like patients who are pregnant, suffering from other diseases, polymedicated or abusing alcohol, smoking, or on drugs. This hinders the identification of undesirable effects that are rare or arise only following long-term exposure, only in a particular group of patients, or because of interactions with other drugs (Singh and Loke, 2012; Alomar et al., 2020). Therefore, it is not surprising that many drug-related risks are discovered only after marketing and that approximately one-third of all such risks are serious enough to be added to the “Warnings and Precautions” section of the package insert, if not even to lead to withdrawal of the drug from the market (Lavertu et al., 2021). For these reasons, pharmacovigilance activities continue for as long as a drug remains on the market by monitoring spontaneous reports and scientific literature and, if necessary, conducting additional clinical (phase IV) and epidemiological studies aimed at better characterizing and quantifying potential risks. All risks identified or suspected during clinical trials and/or drug marketing, as well as any lack of information of clinical relevance regarding the drug’s safety profile, should be appropriately described in the risk management plan and other periodic reports sent to health authorities, together with the measures deemed most appropriate for their monitoring, management and communication to healthcare professionals and patients. If the risks are of modest clinical significance, it is sufficient to present them in the investigator brochure (during clinical trials) and the drug package insert (once on the market), as well as in the periodic reports sent to health authorities. In the case of serious risks, additional risk mitigation and communication measures may need to be put in place, including:

- contraindication to the use of the drug in particular situations or patients (e.g., pregnant women);
- the addition of special warnings and precautions in the package insert;
- urgent communication of information to all investigators (“Dear Investigator” letter) and/or all healthcare professionals (“Dear Healthcare Professional” letter) along with recommendations for risk management;
- changes to the package insert, formulation, or packaging of the drug that are useful in reducing risk (e.g., bottles with child-resistant closures);
- restrictions on drug distribution, such as prescription only by specialists or within programs accessible only to qualified health professionals and patients;
- temporary or permanent discontinuation of clinical trials and/or withdrawal of the drug from the market if, despite all efforts, the risk remains serious, unmanageable, or not justified by the expected benefits.

### 4.2 The precautionary principle

The process described above is perfectly adequate if the risk can be characterized and quantified, so that the most appropriate strategies can be adopted for its management. However, this is not always possible, and there is no shortage of cases where, despite all efforts, major unknowns and doubts remain. This happens particularly often in special situations, such as:

- rare undesirable effects, arising after years of treatment, or affecting special groups of patients (e.g., pregnant, pediatric, or minority ethnic patients) due to the inherent limitations of pre-marketing clinical trials;
- drugs used for the treatment of rare diseases, for which extremely limited safety data are available;
- medicines based on the use of genes, tissues, or cells (known internationally as “advanced therapy medicinal products”) or otherwise on the use of highly innovative and complex techniques for which very limited safety information is available.

In all these cases, the normal approach based on drug risk definition, assessment, and management is no longer sufficient. It is in these situations that pharmacovigilance must consider the “precautionary principle” —a framework for managing risks that are little known and therefore difficult to quantify and predict but potentially devastating, like those that Nassim Nicholas Taleb masterfully described in his *Black Swan Theory* (Taleb, 2007). In fact, there is no universally accepted definition of the precautionary principle, and as late as 1999 there were at least 19 different versions of it (Sandin, 1999). However, UNESCO (2005) summarizes their key elements as follows:

“When human activities are likely to lead to morally unacceptable harm that are scientifically plausible but uncertain, actions should be taken to avoid or mitigate these harms. Morally unacceptable harms are those that threaten human life or health, are severe and irreversible, unfair to present or future generations, or are imposed without regard for the human rights of those affected. The judgment of plausibility must be rooted in appropriate scientific analyses. These analyses should be continuous so that the effects of actions taken can be evaluated. Uncertainty may relate to, but is not limited to, causality or the limits of possible harm. Actions are interventions that are taken before harm occurs and that aim to avoid or reduce harm. Actions should be chosen commensurate with the severity of potential harm, taking into account positive and negative consequences, and with an assessment of the moral implications of both action and inaction. The choice of action should be the result of a participatory process.”

Importantly, the risks involved are not the vague or biased ones that often accompany the development of new technologies—if they were, the precautionary principle would halt progress leading to paralysis. Rather, the moral imperative enshrined in this principle refers to serious and irreversible risks that are judged scientifically plausible after adequate and rigorous analyses but which fail to define their characteristics and probability due to qualitative (e.g., complexity and non-linearity of the system studied, lack of data and/or robust interpretive models) and/or quantitative (e.g., instrumental and methodological) limitations. In such situations, further research may not necessarily reduce the margins of uncertainty, and while it is necessary to continue to evaluate the available data (including assessment of the effects of actions taken), it is imperative to develop a risk prevention or reduction strategy despite persistent uncertainties and complexities. Such an approach must consider the following:

- The attitude to risk of different populations and patients, since the perception of risk and consequently its acceptance varies from person to person and between cultures. Moreover, this attitude is greatly affected by factors such as the importance of the expected benefits, the reversibility of the harm, its familiar or fearful nature, and the possibility of transmission to future generations.
- The foreseeable positive and negative consequences of both the proposed precautionary measures and the lack of them.
- The limitations of the various decision-making models used to assess and manage risks. For example, cost–benefit analysis tends to focus on economic aspects, with a cursory assessment that does not adequately consider the fairness and acceptability of costs and benefits to individual stakeholders.

In practice, in the case of uncertain but likely modest risks, there is usually a tendency to continue data collection and monitoring, informing patients and health professionals of potential risks and recommending caution and reporting any newly emerging information. In the case of potentially serious and irreversible risks, a worst-case scenario approach is taken, intentionally choosing to err on the side of caution rather than default. In borderline situations, where decisions cannot be based solely on logic and facts due to insufficient data, the experience and sensitivity of the individual pharmacovigilance professional become crucial. In such cases, one may pose the fateful question, “If this were my child—or my mother—would I administer this drug? And if so, with what precautions?” Subsequent discussion with colleagues and experts within pharmaceutical companies and health authorities ensures at least partial control of personal biases and errors of judgment at the time of the final decision.

## 5 Justice and its ethical relevance in pharmacovigilance

Like the principle of autonomy, the principle of justice does not stem from the historical foundations of medical ethics and deontology but rather from legal and political thought. It is absent from the Hippocratic Oath and instead appears in a maxim traditionally attributed to Aristotle: “One must treat equals equally and unequals unequally” (Aristotle 1984). However, this precept leaves open the crucial questions of who qualifies as equal or unequal and by what criteria fairness should be applied—questions that have given rise to multiple, often divergent, theories of justice with significant implications for medicine. The idea of human equality—central to any conception of justice—has endured across centuries, shaped by both religious and secular traditions. Christianity, for example, teaches that all humans are equal because they are created in the image and likeness of God (Catechism of the Catholic Church, 2025), while the French Revolution enshrined equality in its motto *Liberté*, *égalité*, *fraternité*. This principle is echoed in Article 1 of the Universal Declaration of Human Rights (United Nations, 1948): “All human beings are born free and equal in dignity and rights.”

In the medical field, the Belmont Report was the first major document to explicitly address justice, particularly distributive justice (National Commission, 1979). It posed foundational questions: who should benefit from medical research? Who should bear its burdens? How can care be distributed equitably, and under what conditions might exceptions be justified? At least six major theories—utilitarian, libertarian, egalitarian, communitarian, and those centered on patient capacity and wellbeing—offer distinct answers to these enduring ethical challenges.

### 5.1 Access to care and equity in clinical research

The implications of these different approaches are vastly different and influence health policy discussion. During the recent COVID-19 pandemic, faced with the exorbitant costs of care needed to save sick anti-vaxxers (Farrenkopf, 2022), it was debated in many countries whether it was fair for these individuals to burden the community (Meer, 2021). Similarly, there are those who question whether it is fair for the community to bear the cost of treating obese, smoking, or alcoholic patients. Answers vary from country to country and person to person, reflecting collective and individual cultural backgrounds. As early as the early 2000s, many citizens in the United States believed that everyone should do everything possible to stay healthy and should also be held financially responsible for their unhealthy behaviors (Wikler, 2004). In Europe, the tendency has prevailed, so far, to ensure that everyone has the necessary medical care, regardless of individual behavior and responsibility, although during the COVID-19 pandemic there was no shortage of critical voices (Capua, 2021). However, it is mainly income that determines the level of access to care, both across countries and across the social strata of the same country. During the recent COVID-19 pandemic, access to vaccines was significantly more limited and delayed in the world’s poorer countries than in developed countries (Duroseau et al., 2023; United Nations News, 2022). These delays were certainly also due to logistical, regulatory, and health organization problems, but what about delays in launching new drugs within the European Union? Here, we are dealing with a bloc of countries with mature healthcare systems, for which the evaluation and marketing authorization is issued at the same time by the EMA; nevertheless, the time between the date of authorization and the date of actual availability to patients varies from 100 days for countries with higher prices and larger markets to 900 days for those with lower prices and smaller markets (Collis, 2022). This is not without consequences: a study conducted in 52 countries around the world clearly shows that delayed access to new therapies significantly reduces longevity in the countries concerned (Lichtenberg, 2005). Criteria of equity should guide not only practice but also clinical research, the risks and inconveniences of which were borne until the first half of the 20th century mainly by the poorest and most vulnerable patients, while the benefits went to the wealthiest (National Commission, 1979). Even today, while scandals such as Tuskegee are no longer imaginable, it remains important to be vigilant about the selection of subjects participating in clinical trials to avoid unwarranted imbalances on racial, national, or census grounds. This is not always easy, given that clinical trials represent the only opportunity for access to advanced care for many patients and poor countries, who are therefore more motivated to participate than those with better means. Not surprisingly, the number of emerging countries participating in international clinical trials has increased many times over in recent years, offering benefits to all parties involved but also raising important ethical issues (The Guardian, 2011). On the one hand, the participation of these countries in global clinical research provides access to advanced therapies and contributes to the scientific development of hitherto marginalized communities, allowing them to enroll the patients needed for clinical trials more quickly at a cost that is also lower than that required in wealthier countries. On the other hand, the condition of need faced by patients in these countries conditions their freedom of choice and thus the value of their informed consent, while the limited resources and experience of local health authorities and ethical committees may lead to inadequate evaluation and supervision of the clinical studies. Finally, international clinical trials do not always address diseases important to local communities, raising important ethical questions in the case of trials conducted in countries that are not the main beneficiaries of the resulting therapies (Weigmann, 2015). Another emblematic case is healthy volunteers participating in phase I clinical trials—the first conducted in humans to assess the tolerability and most frequent undesirable effects of a drug. Despite all the precautions taken, these early studies are particularly risky because of the many unknowns regarding the effects of the new drug, and they obviously cannot bring any health benefits to the healthy volunteer. Undesirable effects are frequent, in rare cases even serious and life-threatening (Attarwala, 2010; Kaur et al., 2016; Lenzer, 2005), and often the study protocol requires performing invasive procedures and confinement for hours or days in dedicated facilities to ensure close monitoring of participants (Abadie, 2010). The only incentive, despite the risks and discomforts, inducing participation in these studies is financial, with fees that a US study a few years ago estimated at between $100 and $300 per day (Edelblute and Fisher, 2015). Given these assumptions, it is not surprising that the majority of healthy volunteers come from the poorest strata of the population and are economically dependent on these studies. Indeed, many of them become “full-time healthy volunteers,” with risks and unknowns for their health—even in the long-term—and for the quality of the data collected (Tishler and Bartholomae, 2003; Fisher and Kalbaugh, 2011). Despite this, there is no centralized registry of healthy volunteers, so the possibility of simultaneous participation in more than one phase I clinical trial cannot be prevented (Walker et al., 2018).

### 5.2 Managing drug risk in an imperfect world

Pharmacovigilance is certainly influenced by the regulatory, cultural, social, and economic conditions of the communities and nations in which it operates. According to the Uppsala Monitoring Centre’s 2022 report, only 15.7% of adverse effect reports in their database were from low- or middle-income countries (Uppsala Monitoring Centre, 2022), which in itself is indicative of the huge gap between developed and undeveloped countries in terms of the maturity and effectiveness of their respective pharmacovigilance systems. Nevertheless, to accomplish its mission, pharmacovigilance must inform and guide clinical research and practice beyond all barriers. This is easier under controlled clinical trial conditions, where all activities and communications aimed at safeguarding study participants and early identification and management of any risks (e.g., study protocols and informed consent forms) are standardized and consistently applied across all participating centers. In this area, the most ethically relevant aspects concern the equitable distribution of the benefits and risks of clinical research among different patient populations and the protection of the rights of participants—particularly the most vulnerable. Therefore, both in pharmaceutical companies and in regulatory bodies (e.g., health authorities and ethics committees), pharmacovigilance experts participate in the planning, conduct, and evaluation of clinical trials, helping to ensure that they are fair and scientifically justified and that all necessary precautions are taken to ensure that the rights of all participants are respected. However, there are special situations in which the principle of justice in the distribution of benefits and risks must be ensured not at a “horizontal” level (e.g., among groups of study participants) but at a “vertical” level. This is the case, for example, with teratogenic or genotoxic cancer drugs, where the important expected benefits for the patient are accompanied by equally important risks for the health of the fetus or future generations. In these cases, more than ever, it is necessary to put in place all possible precautions to mitigate the risk, carefully weigh the benefit–risk ratio for all parties involved (patients and offspring), and adequately communicate available and missing information to physicians, patients, and health authorities to facilitate appropriate therapeutic and regulatory decisions. Things get more complicated once drugs have entered clinical practice; while the number of patients treated and undesirable effects reported can astronomically rise in a matter of months compared to those participating in clinical trials thus providing a much broader basis for risk assessment, other variables come into play which make consistent and equitable risk management much more difficult. The most important among these is certainly related to the maturity and resources of national healthcare systems, including the pharmacovigilance system. Communicating and managing important drug risks requires expertise and resources often accessible only to the wealthiest and most developed countries, and to date few countries have effective risk communication and management strategies in place (Panickar et al., 2022; Bhasale et al., 2021). A case in point of these difficulties involves thalidomide, removed from the market in the 1960s because of the risk of serious congenital malformations if taken during pregnancy. It was allowed back on the market 30 years later for the treatment of multiple myeloma and erythema nodosum leprosum—two serious diseases in which the benefit–risk ratio justifies the use of the drug. In both cases, strict measures are required to avoid use in pregnancy, but these are difficult to implement in countries where leprosy is endemic—typically low- or middle-income. The result is that in countries such as Brazil, cases of phocomelia are increasing (Schuler-Faccini et al., 2007), and thalidomide embryopathy remains a major public health problem. As the ethical principles of pharmacovigilance remain valid in all parts of the world, further efforts are needed from all stakeholders (pharmaceutical companies, health authorities, healthcare providers, and patient associations) to adapt risk management programs to the difficult socioeconomic conditions of developing countries (Mueller and Lewis, 2021), which are often characterized by high rates of illiteracy, crumbling infrastructure, and widespread poverty. We briefly mention here another factor that has a major influence on international pharmacovigilance activities: cultural and religious contexts. Indeed, there is evidence that populations belonging to different cultures and religions show varying readiness to report undesirable effects and follow recommended mitigation measures. It is safe to conclude that different cultures and religions influence how patients experience their relationship with their physicians, tolerate pain, and interpret the information and advice they receive, with obvious implications for risk management (Fainzang, 2010; Yusuf and Wittes, 2017; Keebler et al., 2020). Thus, for pharmacovigilance to be effective, it cannot adopt a single, standardized approach, no matter how rational and rigorous. Rather, it must adapt to the specific conditions of the communities it serves, with due consideration of their socioeconomic, cultural, and religious aspects. Only then can it be effective even in less developed countries, ensuring consistent and equitable management of the risks associated with medicines for all patients worldwide.

## 6 Discussion

In a little over 50 years, pharmacovigilance has made tremendous progress, accompanying the unprecedented development of scientific knowledge and the resulting rapid increase in available drug therapies. Born after the thalidomide disaster out of the ethical imperative to safeguard public health, pharmacovigilance has grown over time, equipping itself with increasingly sophisticated standards, methods, and infrastructure, with the ambition of ensuring the best benefit–risk ratio for every drug for all patients worldwide. The path has not been without errors and scandals (often attributable to the malice or inexperience of a few), and it remains arduous for a whole host of theoretical and practical reasons. However, there is no doubt that pharmacovigilance represents, to date, one of the pillars of any evolved healthcare system, and this role is likely to grow even more as increasingly innovative therapies and interventions are developed that pose unprecedented challenges. A recent example is the COVID-19 pandemic, which highlighted the need to manage the global risks and unknowns posed by vaccines produced with a technology—messenger RNA—never before authorized for use in clinical practice. Additional challenges are related to the current development of:

- advanced therapy medicinal products (ATMPs) based on genetic, cellular, or tissue engineering techniques;
- bionic organs, intended to restore impaired sensory or motor functions;
- digital therapies—software that has been clinically assessed and approved for use in humans to treat a wide range of diseases.

In all these cases, highly innovative approaches promise important results in the treatment of serious diseases and disabilities. However, at the same time, they present potential risks that are difficult to manage because of the unknowns and peculiarities associated with new techniques. Consider, for example, the duration of the expected effect: chemical and biological drugs, after a period that varies according to the half-life of the molecule (usually days or weeks), are eliminated from the body and stop exerting their effects, whether positive or negative. In contrast, many gene therapies involve a single administration of the correct gene, which is integrated into the individual’s genetic makeup and can be passed on to his or her offspring. This difference alone would suffice to explain the increasingly important role that pharmacovigilance will be called upon to play in public health in order to anticipate and prevent unknown and potentially irreversible undesirable effects in treated patients and future generations. Likewise, adverse effects of pharmaceuticals beyond the treated patient are increasing, driven by the growing presence of persistent and pseudo-persistent compounds in ecosystems via excretion, manufacturing waste, and improper disposal (UNEP, 2019). These substances pose significant, long-term risks to both biodiversity and human health, ranging from catastrophic declines in vulture populations due to diclofenac (Richards et al., 2017; Frank and Sudarshan, 2024) to impaired reproduction in fish caused by 17α-ethinylestradiol (Salierno and Kane, 2009) and the promotion of antimicrobial resistance (Haenni et al., 2022). Pharmaceuticals containing per- and polyfluoroalkyl substances (PFAS) represent another prime example of this emerging threat (Fenton et al., 2021; Johnson et al., 2020; Ruyle et al., 2025), necessitating an urgent expansion of traditional pharmacovigilance into ecopharmacovigilance. This aligns with the World Health Organization’s inclusive definition of pharmacovigilance, which extends beyond direct patient harm to encompass any possible drug-related problems. As an emerging discipline, ecopharmacovigilance seeks to identify, assess, and mitigate the environmental and public health risks associated with pharmaceutical agents (International Society of Pharmacovigilance, 2025). However, ecopharmacovigilance currently faces major systemic and operational challenges, including the lack of comprehensive and harmonized data on the environmental occurrence and effects of pharmaceuticals (Uppsala Monitoring Centre, 2025), the limited scope and weight of relevant regulations (Kittery and Miettinen, 2023), and the limited transparency of environmental risk assessment dossiers (Oelkers and Floeter, 2019). Addressing these gaps requires a systems thinking approach, prioritizing harmonized international guidance, interdisciplinary collaboration, and the routine linkage of environmental monitoring with drug safety systems (Witte and Müller, 2025; Gómez-Oliván, 2019). In addition to the challenges posed by scientific innovation and environmental contamination, there are operational challenges related to the availability of resources (especially in low- and middle-income countries), the steady increase of drugs being studied and on the market, the constant acceleration of drug development and regulatory assessment (e.g., fast-track procedures), and the increasing complexity of standards and procedures that ongoing deglobalization makes less and less internationally harmonized. In response to these challenges, pharmacovigilance continues to evolve by developing equally innovative tools and methods, with a pronounced use of digital solutions for extracting real-world data, such as derived from EHR and social media rather than randomized clinical trials. Digital solutions are also being proposed to collect clinical data of interest automatically and continuously (e.g., using sensors coupled with a cell phone app) to report adverse effects and to improve the efficiency of risk management programs (Da Silva-Tillmann et al., 2022; Hopf et al., 2014; Lavertu et al., 2021). Finally, the very recent development of artificial intelligence techniques such as natural language processing, text mining, and machine learning, promises to revolutionize pharmacovigilance by enabling the automatic extraction and processing of vast amounts of data even from unstructured sources, with the possibility of automating the identification and prediction of possible risks (Wong et al., 2018; Basile et al., 2019; Bate and Hobbiger, 2021; Trifirò and Crisafulli, 2022). Nevertheless, many critical decisions in pharmacovigilance remain a matter of judgment and thus require ethical thinking. In addition, amidst the fervent pursuit of scientific and technical solutions to the “hows” of 21st-century pharmacovigilance, a host of ethical questions concerning the “whys” of this discipline have regrettably been neglected and remain under-investigated. Here, computerized algorithms, norms, and standard procedures are of little help; it would be more useful to address these issues by re-focusing on critical thinking and bioethics; the four principles of Beauchamp and Childress (2019) may be a good starting point in this regard. As already mentioned, their proposed method has its limitations, and in the event of conflict between the four principles, it becomes necessary to weigh one against the other by seeking a meeting point acceptable to all parties involved—a meeting point that will inevitably be influenced by the cultural context of reference. However, this approach seems far preferable to a rigidly defined hierarchy of values, perhaps accompanied by a decision tree. Thus, if we look at pharmacovigilance from the standpoint of principlism, there is no doubt that the *prima facie* moral duty of this discipline remains that of non-maleficence, according to the motto *primum non nocere* applied to medicines. However, there is no shortage of situations in which the proper protection and consideration of other principles must be placed alongside this guiding principle. Some examples follow.

Autonomy. Patients are undoubtedly the main beneficiaries of pharmacovigilance activities, and at the same time the source of almost all clinical information regarding the undesirable effects of drugs, the only exception being healthy volunteers enrolled in phase I clinical trials. It is therefore clear that they should be involved in managing the related risks through appropriate and balanced communication strategies, as advocated in 1997 by the visionary Erice Declaration on the Communication of Information Concerning Drug Safety (Springer Nature, 1997). Such strategies should ensure adequate feedback to those patients reporting undesirable effects and complete information regarding precautions to be taken. In addition, they should consider the cultural, linguistic, social, and religious aspects of the patient, modulating the message in a way that optimizes understanding. Greater effort in this regard could promote adherence to prescribed therapy, active and informed patient involvement in risk management, and increased public trust in health authorities and pharmaceutical companies. Unfortunately, 28 years after the Erice Declaration, this remains largely unrealized, and respect for patients as equal partners in drug risk management persists as a major ethical issue to be answered by pharmacovigilance.

Beneficence. Drawing inspiration from the doctrine of double effect developed by the scholastic philosopher and theologian Thomas Aquinas, any drug used appropriately can be ethically justified, since of its two potential effects (beneficial and harmful), only the former are desired, while the latter are unintended. Nevertheless, the assessment of the benefit–risk ratio remains critical to ensuring the optimal and ethical use of drugs, with all related challenges and unknowns. In addition, there are critical issues caused by two strongly dated structural elements that are no longer adequate for public health interests.

The first issue concerns the binary decision-making model on which rests the entire regulatory scaffolding concerning the clinical development of new drugs and their evaluation by health authorities. Under this model, a drug is declared sufficiently safe and effective for use in clinical practice the moment it receives marketing authorization. This is patently risky given the limitations of pre-registration clinical trials. Therefore, a paradigm shift would be useful through, for example, the adaptive licensing approach (Eichler et al., 2012). This model recognizes the unknowns present at the end of clinical development and overcomes the dichotomy of the current regulatory process regarding marketing authorization by moving to a continuous process, with iterative steps of data collection, evaluation, and clinical-use authorization in parallel with the growing wealth of knowledge about the drug. In addition to handling, much better than the current model, the limitations of the evidence gathered during clinical trials, adaptive licensing could play an important role in reducing the off-label use of established drugs (i.e., those well-known and commonly used in clinical practice) in indications or patient populations not approved by health authorities only because they have not been evaluated in pre-registration clinical trials. Pharmacovigilance can and should certainly support this step change by making full use of the sophisticated methods and techniques available today.

The second critical issue concerns the exclusive nature of pharmacovigilance-related assessments and interactions, which to date have involved mainly two actors: health authorities and pharmaceutical companies. Serious safety problems with marketed drugs have been repeatedly detected by independent researchers. However, they usually do not have access to most data available to pharmaceutical companies and health authorities for reasons related to the protection of intellectual property and the prevention of abuse by competing companies. While respecting these legitimate concerns, as early as 2012 the Committee on Ethical and Scientific Issues in Studying the Safety of Approved Drugs of the Institute of Medicine asked the US FDA to initiate a pathway to determine how to “…appropriately balance the interests of public health, privacy, and data owners to facilitate the disclosure of relevant information” (Mello et al., 2012). Independent experts, the medical community, and patients are still waiting.

Justice. Despite tremendous progress in recent years and important contributions to public health protection, pharmacovigilance still remains a luxury reserved for wealthy countries that have the expertise and resources to manage it. In much of the world, however, the sequence of activities from information collection, processing, and communication to drug risk governance itself is lacking. Thus, while in more developed countries patients and health professionals can rely on sophisticated additional measures for risk communication and mitigation, in less developed countries the package insert remains the only means available to inform patients—with predictable consequences given the high levels of illiteracy. One hope for greater equity in drug risk management may come from the use of innovative digital tools, enabling, for example, the transmission through cell phones of user-friendly videos and educational material, taking advantage of the widespread dissemination of these media even in poorer countries (Zhang, 2018; Silver, 2019). A further opportunity lies in the strict application of ethical principles to pharmacovigilance activities conducted in countries that do not yet have a mature regulatory system, thus leading to the progressive improvement of standards on a cultural before a legal level. Alongside the challenges for pharmacovigilance posed by international socioeconomic inequalities, one should not overlook those concerning disadvantaged ethnic groups and social classes within the same country—as seen, for example, in the disturbing case of full-time healthy volunteers.

In conclusion, pharmacovigilance was born out of the ethical imperative to “do no harm” with medicines intended to cure. Over the years, it has grown from a semi-unknown and poorly codified discipline to a pillar of every evolved healthcare system. An immense and ever-growing number of rules, laws, and regulations now prescribe its activities, with sophisticated tools increasing its capabilities and efficiency every day. However, while most useful in meeting the operational challenges of pharmacovigilance, these measures prove inadequate for addressing the many ethical issues concerning the benefit–risk ratio of therapies both during research and in clinical practice. Moreover, they risk reducing the role of drug safety professionals to mere executors of processes whose meaning and implications they no longer understand. Therefore, alongside the necessary technical and regulatory developments, it is essential for pharmacovigilance to reaffirm its ethical roots and fortify them with the study and application of moral principles that are too often taken for granted.
